# The Quantity and Quality of African Children's IgG Responses to Merozoite Surface Antigens Reflect Protection against *Plasmodium falciparum* Malaria

**DOI:** 10.1371/journal.pone.0007590

**Published:** 2009-10-27

**Authors:** David Courtin, Mayke Oesterholt, Harm Huismans, Kwadwo Kusi, Jacqueline Milet, Cyril Badaut, Oumar Gaye, Will Roeffen, Edmond J. Remarque, Robert Sauerwein, André Garcia, Adrian J. F. Luty

**Affiliations:** 1 Medical Microbiology, Radboud University Nijmegen Medical Centre, Nijmegen, The Netherlands; 2 Department of Parasitology, Biomedical Primate Research Centre, Rijswijk, The Netherlands; 3 Unité de Recherche (UR) 010 « Santé de la mère et de l'enfant en milieu tropical », Institut de Recherche pour le Développement, Université Paris Descartes, Paris, France; 4 Laboratoire de Parasitologie et de Mycologie, Département de Biologie et d'Explorations fonctionnelles, Faculté de Médecine, Université Cheikh Anta Diop, Dakar, Sénégal; Walter and Eliza Hall Institute of Medical Research, Australia

## Abstract

**Background:**

Antibodies, particularly cytophilic IgG subclasses, with specificity for asexual blood stage antigens of *Plasmodium falciparum*, are thought to play an important role in acquired immunity to malaria. Evaluating such responses in longitudinal sero-epidemiological field studies, allied to increasing knowledge of the immunological mechanisms associated with anti-malarial protection, will help in the development of malaria vaccines.

**Methods and Findings:**

We conducted a 1-year follow-up study of 305 Senegalese children and identified those resistant or susceptible to malaria. In retrospective analyses we then compared post-follow-up IgG responses to six asexual-stage candidate malaria vaccine antigens in groups of individuals with clearly defined clinical and parasitological histories of infection with *P. falciparum*. In age-adjusted analyses, children resistant to malaria as well as to high-density parasitemia, had significantly higher IgG1 responses to GLURP and IgG3 responses to MSP2 than their susceptible counterparts. Among those resistant to malaria, high anti-MSP1 IgG1 levels were associated with protection against high-density parasitemia. To assess functional attributes, we used an *in vitro* parasite growth inhibition assay with purified IgG. Samples from individuals with high levels of IgG directed to MSP1, MSP2 and AMA1 gave the strongest parasite growth inhibition, but a marked age-related decline was observed in these effects.

**Conclusion:**

Our data are consistent with the idea that protection against *P. falciparum* malaria in children depends on acquisition of a constellation of appropriate, functionally active IgG subclass responses directed to multiple asexual stage antigens. Our results suggest at least two distinct mechanisms via which antibodies may exert protective effects. Although declining with age, the growth inhibitory effects of purified IgG measurable *in vitro* reflected levels of anti-AMA1, -MSP1 and -MSP2, but not of anti-GLURP IgG. The latter could act on parasite growth via indirect parasiticidal pathways.

## Introduction

IgG antibodies' pivotal role in anti-malarial protection was demonstrated by seminal studies involving the passive transfer of IgG, purified from sera of semi-immune adults, to non-immune patients resulting in clearance of parasitaemia [1], [2], [3]. This protection reflects antibody responses directed to blood stage antigens of *P. falciparum*, although the precise mechanism(s) involved remains unclear. Specific IgG are proposed to have either a direct [4], [5], [6] and/or indirect effect [7] on parasite growth inhibition. Among the IgG subclasses, IgG1 and IgG3 are thought to play a key role in the protection [8], [9]. It is believed that these subclasses can neutralize parasites directly, by inhibiting parasite invasion or growth in erythrocytes, or indirectly by a mechanism involving cooperation between parasite-opsonizing antibodies and monocytes through binding to the Fcγ receptor IIA, leading to secretion of soluble parasite growth-inhibitory factors such as nitric oxide or tumor necrosis factor-alpha [7], [10], [11]. In the latter case the cytophilic IgG subclasses IgG1 and IgG3 are thought to be of paramount importance [10], [11].

Defining immune surrogates or, even better, correlates of protection is considered an essential step in the rational development of malaria vaccines and sero-epidemiological studies are one of the valuable tools with which putatively protective anti-malarial antibody responses can be identified. Here, we present the results of a sero-epidemiological study in the Niakhar area in Senegal in which parasitological, clinical and epidemiological data were collected during one-year of close, active follow-up of a large cohort of children and adolescents. Plasma samples, used for antibody assessments, were collected from participants at the end of the study period. The objectives we set were 1) to identify groups of individuals based on clearly defined differences in their capacity to control infection and/or disease due to *P. falciparum*, 2) to retrospectively assess the association between anti-malarial protection and IgG antibody isotype responses to a panel of asexual stage antigens representing the foremost vaccine candidates, and 3) to use functional assays of antibody activity *in vitro* to determine their predictive value for the results of the sero-epidemiological study.

## Materials and Methods

### Study site, study design and blood collection

The study took place during the whole of 2003 in Diohine and Toucar, two villages located 6 km apart in the Niakhar district situated 135 km south-east of the capital, Dakar. The study area, study design, and local epidemiology of malaria have been described in detail elsewhere [12]. Briefly, in this area, malaria is characteristically seasonal but stable, with an inoculation rate estimated at 9–12 infective bites per person per year. Transmission occurs predominantly during the rainy season, between September and December, and is due exclusively to mosquitoes of the *Anopheles gambiae s.l.* complex [13]. The study design included repeated cross-sectional surveys, to identify sub-clinical parasitaemias, conducted in both non-transmission (January, April, June), and transmission (September, October, December) seasons, when thick blood smears (TBS) were prepared according to standard protocols. On the Giemsa-stained TBS, the number of parasites was counted in 50 high-power fields. The parasite density (PD), defined as the number of parasites per 100 leucocytes, was then determined by dividing the mean number of parasites by the mean number of leucocytes per field. The latter was assessed on 30 standardised microscopic fields. A TBS was declared negative when no parasites were detected in 200 fields. Active surveillance to detect malaria attacks was conducted during the transmission season (September to December 2003). During this period, trained primary health care personnel visited all study participants twice a week to check axillary temperature and to assess their clinical status. To be included in the study, children or young individuals (less than 20 years old) and their parents had to be present in study area (Niakhar) during the follow-up. Parents were invited to bring their child to the dispensary in case of fever at any time. When a diagnosis of malaria was suspected for any reason and on any occasion, a TBS was performed and a questionnaire related to clinical signs completed. Individuals were given anti-malarial therapy according to the recommendations of the Senegalese National Control Program for malaria at that time (i.e. first-line treatment with chloroquine). After the transmission season and at the end of the one-year parasitological and clinical follow-up (December 2003), plasma samples were isolated from venous blood collected from 305 individuals aged 7 to 19 years old.

The study was explained in detail to all participants and their parents, and either they or their parents gave their signed informed consent. The ethics committee of the Health Ministry of Senegal approved the study protocol (N°000526/MS/DERF/DER).

### Segregation of children according to malariometric data

#### Uncomplicated malaria attack (UMA) group

We defined uncomplicated malaria attacks (UMA) as the association of an axillary temperature greater than 37.5°C with a PD equal to or higher than 2,500/µl and with no other apparent cause of fever, in order to avoid potential bias. Any individual identified as having had at least one UMA during the follow-up period was included in the UMA group.

#### Asymptomatic carriage (AC) groups

Individuals with no UMA during the follow-up and with at least one parasite-positive TBS were considered as asymptomatic carriers (AC). All TBS performed in the 15 day period immediately following anti-malarial treatment were excluded when assessing the study participants' parasitological phenotype. The AC group was further segregated according to their levels of parasitaemia (low vs high). As a UMA was defined as the association of fever with a parasite density equal to or higher than 2500/µl, we selected the same threshold to discriminate between two AC groups. The AC group with low parasitaemia (ACLP) group thus comprised individuals who had at least one parasite-positive TBS but with a PD below 2,500/µl on each occasion. The AC group with high parasitaemia (ACHP) comprised individuals who had at least one positive TBS during the follow-up but with a PD equal to or above 2,500/µl on at least one of the parasite-positive TBS. This sub-segregation was designed to allow evaluation of antibody response(s) potentially associated with anti-parasite (ACLP vs ACHP groups), anti-disease (ACHP vs UMA groups) and/or combined anti-parasitic/anti-disease (ACLP vs UMA groups).

#### Uninfected individuals

Children with no UMA during the active follow-up and no *P. falciparum* parasites on any of their six TBS could be considered to either be completely protected or simply not exposed to infection. To avoid possible confounding we excluded all such individuals (n = 33) from all analyses.

### Antibody measurements and *HBB* AS genotype

An Enzyme-Linked Immuno-Sorbent Assay (ELISA) following a standardized methodology described in the Afro-immunoassay network standard operating procedure (procedure number AIA-001-02) was used to assess the antibody response, at the end of the follow-up (December 2003), to the following panel of recombinant proteins derived from sequences of asexual stage antigens of *P. falciparum*:

1. MSP1_19 20–43, 1615–1723_/Uganda-Palo-Alto strain
2. MSP2/3D7 & MSP2/FC27
3. MSP3 _161–276_/FC27 strain
4. AMA1 _25–545_/FVO strain
5. GLURP _25–514_/F32 strain

MSP1_19_ (Pasteur Institute, Paris, France) was expressed in a Baculovirus/insect cell system [14], AMA1 (Biomedical Primate Research Centre, Rijswijk, The Netherlands) in *Pichia pastorius* [15], and MSP2/3D7, MSP2/FC27 (both La Trobe University, Melbourne, Australia), MSP-3 and GLURP R0 (both Statens Serum Institute, Copenhagen, Denmark) all in *Escherichia coli* [16]. ELISA plates were coated with 100 µl of recombinant protein solutions (1X PBS) at a final concentration of 1 µg/ml. 150 µl of blocking buffer (3% milkpowder in PBS - 0.1% Tween 20) was added and kept at room temperature for 1 hour. Plasma samples were diluted in dilution buffer (1% milk powder in 1X PBS 0.1% Tween 20, 0.02% NaAz) 1∶200 for total IgG (IgGt) and 1∶50 for IgG1, IgG2, IgG3 and IgG4 for all recombinant proteins except for AMA1 for which dilutions used were: 1∶2000 (IgGt) and 1∶500 (IgG1, IgG2, IgG3 and IgG4). The monoclonal antibodies used for determination of the immunoglobulin isotypes were mouse anti-IgG1 (clone NL16, Skybio), anti-IgG2 (clone HP 6002, Sigma), anti-IgG3 (clone ZG4, Skybio) and anti-IgG4 (clone RJ4, Skybio). Two polyclonal antibodies conjugated to HRPO were used: a goat anti-human IgG (gamma) (Caltag) diluted 1∶3000 for IgG and a goat anti-mouse IgG (H+L) (Catltag) diluted 1∶2000 for IgG1, 1∶5000 IgG2 and IgG3, and 1∶3000 for IgG4. Bound enzyme was detected with TMB and the reaction was stopped with 0.2 M H_2_SO_4_ (100 µl/well). Plates were extensively washed between each incubation period with PBS Tween-20 (0.1%) NaCl (0.5 M). The optical density (OD) was read at 450 nm (reference filter 620 nm). Positive-control plasma samples from Gabonese individuals and negative-control plasma samples from Dutch individuals were included in each plate and results were expressed in arbitrary units (AU) calculated from the formula: 100× [ln(OD test plasma)−ln(OD negative control plasma)]/[ln(OD positive control plasma)−ln(OD negative control plasma)] [17]. The positivity thresholds were determined from the mean reactivities +2 SD of 30 plasma samples from Dutch non-immune volunteers. Since it is known that antibodies of the IgG2 and IgG4 isotypes with specificity for the asexual stage antigens assessed here are generally present at lower prevalence and intensity compared with cytophilic isotypes, they were quantified, in a first step, on a sub-set of 42 samples. If the proportion of responders among these samples, for either isotype, was >30% (an arbitrarily chosen threshold), we proceeded to determine the corresponding responses in samples from the whole study population.

Previous studies have shown that carriage of the sickle cell trait is associated with protection against uncomplicated malaria and with the modulation of anti-parasite antibody responses [18], [19], [20], [21], [22], therefore all the individuals carrying the *HBB* AS genotype (n = 32) were excluded from analyses that compared antibody responses between groups. The determination of the different *HBB* genotypes was performed by PCR/RFLP as previously described [23].

### Antibody purification for functional assay

Thirty plasma samples, matched according to age, gender and area of residence, were randomly selected from each of the ACLP, ACHP and UMA groups. IgG was purified from 600 µl of each of these plasma samples using SpinTrap Protein G columns (GE, Eindhoven, The Netherlands) using procedures recommended by the manufacturer. The purified IgG samples were concentrated using Vivaspin 20 ultrafiltration spin columns (Sartorius, Palaiseau, France) according to the recommended protocol to a final concentration of at least 20 mg/ml and the concentrated IgG were then filter-sterilized using 0.22 µm centrifuge filters (Millipore, Carrigtwohill, Ireland). The IgG were quantified using the NanoDrop® ND-1000 spectrophotometer and the required IgG concentration for the parasite growth assay (20 mg/ml) was obtained through appropriate dilution in RPMI 1640 medium before each functional assay.

### Parasite Growth Inhibition Assay

Parasite Growth Inhibition (PGI) was performed as previously described with minor modification [24]. Briefly, 3D7 strain *P. falciparum* parasites maintained in culture *in vitro* were synchronized using alanine treatment and collected for use at the beginning of schizogony. Assays were initiated in flat-bottomed 96-well tissue culture plates with a starting parasitaemia of 0.3% in a final volume of 100 µl containing 10% normal human serum, 20 µg/ml of gentamicin, a final concentration of test IgG of 10 mg/ml (50 µl of the 20 mg/ml IgG preparation) at a haematocrit of 2% in RPMI 1640. The growth inhibitory capacity of each sample was assayed in triplicate and each assay plate included positive (purified rabbit anti-AMA1 IgG) and negative control samples (RBC alone and parasite culture without IgG). Assays were collected for analysis 42–44 h after initiation and the PGI in the presence of IgG was assessed by measuring the *Plasmodium* lactate dehydrogenase levels in parasite cultures [25]. The PGI was calculated as follows:
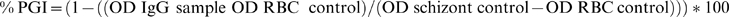


### Statistical analyses

The association of specific IgG responses with anti-malarial protection was first assessed using a linear model (ANCOVA) taking into account the age effect on the IgG levels. The comparison of IgG levels between the UMA and AC groups was performed to investigate the potential anti-malarial protective responses and the comparison of the IgG levels between the UMA vs ACHP, ACHP vs ACLP, UMA vs ACLP groups aimed to identify more precisely anti-parasite and/or anti-malarial (clinical) protective responses.

Logistic regression was applied for the analysis of associations with protection against malaria attacks (UMA vs AC groups) which was used as the dependent variable against the explanatory variables (IgG responses/age) shown to be associated by ANCOVA. This approach allowed for assessment of the independence of associations of IgG responses with protection in the linear model and to additionally check for the presence or absence of interactions between putatively protective IgG responses. The relationship between the number of antigens recognized at high titre by each individual and group (UMA, ACHP and ACLP) was also investigated using logistic regression. For this purpose, antibody titres (IgGt responses to AMA1 and MSP1, IgG3 to MSP3 and MSP2 [average of responses to the FC27 and 3D7 alleles], and IgG1 to GLURP) in the whole study population were re-coded into tertiles and values in the top tertile were considered a high response for each specific antigen. Next, the number of high responses was calculated per individual. Logistic regression was then performed with group (UMA, ACHP and ACLP) as outcome and age and number of antigens recognized at high titre as explanatory variables.

The non-parametric Kruskal Wallis and Mann-Whitney tests were used to test for differences in levels of PGI and antibody levels between the different groups. All analyses were carried out in STATA version 8 (StataCorp, College Station, TX).

## Results

### Demographic and other characteristics of study participants

Details of the study participants are presented in Table 1. Their mean age was 11.8 years and the sex ratio (female∶male) was 0.68. 178 were inhabitants of Diohine and the other 127 were from Toucar. The mean age of those in whom one or more uncomplicated malaria attacks (UMA group) were detected was significantly lower than that of those in whom asymptomatic carriage of parasites (AC group) was detected, while the gender and village distribution of the two groups was similar (Table 1). *HBB* AS carriers were significantly less frequent in the UMA compared to the AC group. After exclusion of those with *HBB* AS, the UMA group for analyses comprised 89 children among whom 64, 21 and 4, had had, respectively, 1, 2 and 3 malaria attacks during the follow-up period. The AC group for analyses comprised 157 children (with sub-groups defined thus: 89 asymptomatic carriage with low parasitemia [<2500/ul, ACLP] & 68 asymptomatic carriage with high parasitemia [≥2500/ul, ACHP]).

**Table 1:** Participants' characteristics.

	n	Age*	Gender**	Villages***	RBC defect
		(Range, IQRs)	Female/Male	Diohine/Toucar	*HBB* AS carriers****
Whole population	305	11.8 (7–19, 9–14)	123/182	178/127	32
Groups†					
UMA	94	10.9 (7–18, 9–12)	36/58	53/41	5
AC	178	12.0 (7–19, 10–14)	70/108	114/64	21
ACHP	74	11.5 (7–19, 9–14)	31/43	51/23	6
ACLP	104	12.4 (7–17, 11–14)	39/65	63/41	15

UMA = uncomplicated malaria group; AC = asymptomatic carrier group; ACHP = asymptomatic carrier subgroup with high parasitemia; ACLP = asymptomatic carrier subgroup with low parasitemia; RBC = red blood cells.

* Significant age difference between UMA, AC and uninfected groups (Anova, p<0.0001).

** No significant gender difference in the groups (Chi2, p = 0.513).

*** No significant difference between groups (Chi2, p = 0.25).

**** The Hbb AS genotype was significantly less frequent in the UMA group than the group of individuals free of clinical malaria (Chi2, p = 0.049).

† Excludes the uninfected group (33 individuals).

### Antibody profiles

**Figure 1:**
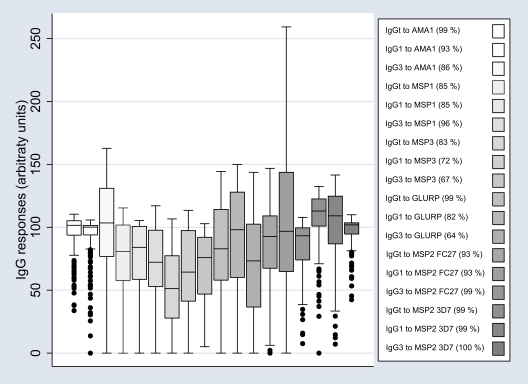
IgG antibody levels in all participants. Total and cytophilic IgG subclass (IgG1 & IgG3) levels of 273 individuals (*HBB* AS carriers excluded) with specificity for the panel of asexual blood stage antigens of *P. falciparum*. Box-whisker plots represent medians with 25^th^ and 75^th^ percentiles (boxes), and with 10^th^ and 90^th^ percentiles (whiskers), outliers as discrete dots. The prevalence of positive responders to each recombinant proteins is noticed between brackets. A positive responder was defined as an individual who had a level of specific IgG over the positivity thresholds. These positivity thresholds were determined, for total IgG, IgG1 and IgG3 to each recombinant proteins, from the mean reactivities+2 SD of 30 plasma samples from Dutch non-immune volunteers.

**Figure 2:**
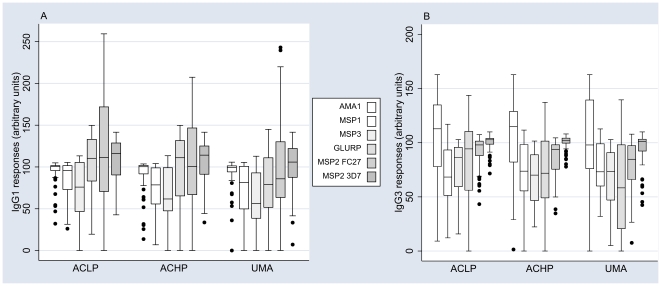
IgG antibody isotype responses in groups of children with and without malaria attacks. IgG1 (A) and IgG3 (B) responses to a panel of recombinant proteins corresponding to 5 different *P. falciparum* asexual stage antigens in groups of children segregated according to their status as either low (ACLP, <2500 parasites/ul) or high (ACHP, ≥2500 parasites/ul) asymptomatic parasitemia carriers, or those with one or more malaria attacks (UMA, parasitemia plus fever) during 12 months' follow-up. Box-whisker plots represent medians with 25^th^ and 75^th^ percentiles (boxes), and with 10^th^ and 90^th^ percentiles (whiskers), outliers as discrete dots.

**Figure 3:**
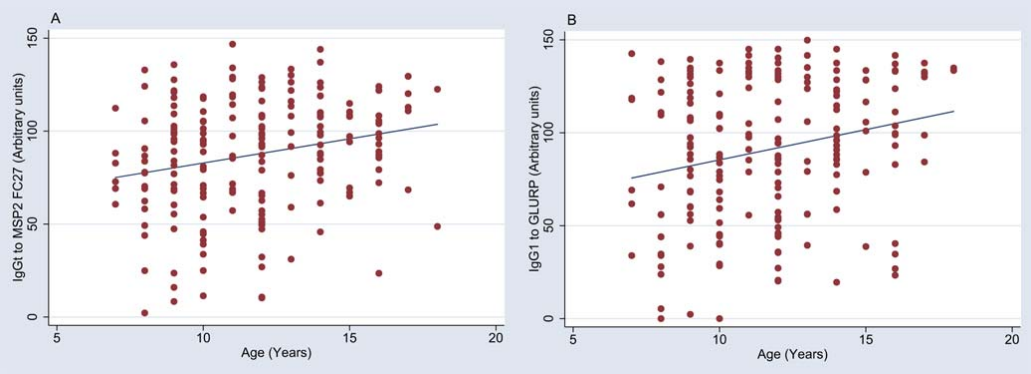
Age-dependent changes in antibody responses. Graph was performed with the regression fit command of Stata software. The age effect on IgGt to MSP2 FC27 and IgG1 to GLURP was significant (ANCOVA, table 3, ACLP vs UMA, p = 0,046 and p = 0,047 respectively).

A high proportion of the study participants had IgG responses to all recombinant proteins. Cytophilic IgG1 and IgG3 isotype-specific responses were most frequent, with prevalences ranging from 64% (IgG3 to GLURP) up to 100% (IgG3 to MSP2 3D7)(Fig. 1). IgG1 and IgG3 responses segregated according to study participants' clinical/parasitological history are presented in Figure 2. In general, regardless of the antigen or IgG isotype, the lowest responses were observed in the UMA group. A number of potentially confounding factors were assessed: (i) *P. falciparum* parasitemia at the time of the blood draw in December 2003 (47% were carrying infections, as determined by microscopical examination of blood smears) did not significantly influence the level of IgG responses (data not shown); (ii) the proximity of malaria episodes to blood draws in the UMA group (2 individuals had a malaria episode in December, 13 in November) did not affect antibody levels (data not shown). Age had an effect on the levels of specific antibody responses, most of which increased with age. Particularly notable was IgG1 with specificity for GLURP and IgGt with specificity for MSP2 FC27 (Fig. 3). Given that it was clearly related with antibody levels, we included age as a continuous variable in all linear analyses.

### Protection against malaria

The putative protection against uncomplicated *P. falciparum* malaria conferred by carriage of *HBB* AS [26] was confirmed here (Table 1), showing that our study had sufficient statistical power to detect the effects of factors that have proven associations with anti-malarial immunity. In order to investigate potential associations between antibody responses and protection against malaria attacks, we therefore first used a linear model (Analysis of covariance (ANCOVA)) to compare the levels of specific IgG between the UMA and unsegregated AC groups (Table 2). This analysis revealed that the levels of IgG3 directed to either of the MSP2 antigens (FC27 and 3D7 allelic forms) and the levels of IgG1 directed to GLURP were significantly higher in the AC compared to the UMA group (Table 2). Furthermore, a similar trend, but of only borderline significance, was found for IgG3 with specificity for GLURP. No such associations were observed for IgG responses to either AMA1, MSP1 or MSP3.

**Table 2:** Associations between IgG responses and protection against malaria attacks.

			Linear model (ANCOVA)**	Logistic regression†
IgG responses*	Status	IgG mean (AU)	Coefficient	P	CI	P	OR
MSP2 3D7 IgGt	UMA/AC	105.34/112.88	7.25	0.005	[2.19–12.31]		
MSP2 3D7 IgG3	UMA/AC	95.13/100.25	5.14	<0.0001***	[2.51–7.75]	0.049	0.70
MSP2 FC27 IgGt	UMA/AC	79.88/93.61	12.56	0.002	[4.60–20.53]		
MSP2 FC27 IgG3	UMA/AC	80.15/90.14	9.37	<0.0001***	[4.78–13.95]	0.076	0.84
GLURP IgGt	UMA/AC	72.44/91.44	16.99	<0.0001***	[7.84–26.15]		
GLURP IgG1	UMA/AC	80.45/100.54	17.64	<0.0001***	[7.83–27.45]	0.024	0.91
GLURP IgG3	UMA/AC	59.47/77.73	16.28	0.003	[5.49–27.08]		

* Presented are only those antibody responses, from the 20 tested, found to be significantly associated with malariometric status (P<0.05 before multiple test correction); IgGt: total IgG.

** The effect of malariometric status on IgG responses, determined by ANCOVA, was adjusted for age.

*** Significantly different after adjustment for multiple tests (Bonferroni correction, threshold of significance: P = 0.002).

† Logistic regression was used to assess associations with protection against malaria attacks, where status (UMA vs AC groups) was used as the dependent variable against the explanatory variables (IgG subtype responses/age) shown to be significantly associated by ANCOVA. OR values were assessed for 10 AU increased.

Note: age was also independently associated with malaria protection in this analysis (P = 0.034, OR = 0.89).

The logistic regression model confirmed the apparent anti-malarial protective effects of the IgG isotype responses identified by ANCOVA. Thus, the levels of IgG3 with specificity for MSP2 3D7, and of IgG1 with specificity for GLURP were both shown to be significantly higher in the AC versus UMA groups, while a similar trend, but of borderline significance, was found for IgG3 directed to the FC27 allele of MSP2. Independently of the IgG activity, age (p = 0.034, OR = 0.89) also differed significantly between the groups. Possible interactions between the three antibody responses were investigated to determine potential combination effects. No interaction was observed in the logistic regression model, indicating that the three responses act independently, and, possibly, additively.

### Protection against disease and/or parasitaemia

The sub-segregation of the AC group (ACHP and ACLP) was designed to allow for assessments of possible associations between specific IgG responses and different aspects of acquired immunity. The comparison of IgG levels between the ACHP and UMA groups, respectively resistant and susceptible to the clinical symptoms associated with *P. falciparum* infection, thus aimed to determine whether an ‘anti-disease’ type of immunity was associated with any of the antibody activity measured here. By ANCOVA, the levels of IgGt to both MSP2 (3D7 allelic form) and to GLURP, of IgG1 to GLURP and of IgG3 to MSP2 (both allelic forms) were found to be higher in the ACHP compared to the UMA group (Table 3), but after multiple test adjustments none of these associations remained statistically significant, and further logistic regression analysis was therefore not performed.

**Table 3:** Associations between IgG responses and anti-disease/anti-parasite immunity.

			ANCOVA*	Logistic regression***	
			Status		
IgG responses	Status	IgG mean (UA)	Coefficient	P	CI	P	OR
IgGt to MSP2 3D7	UMA/ACHP	105.34/112.48	7.04	0.039	[0.34–13.74]		
IgG3 to MSP2 3D7		95.13/100.03	4.93	0.008	[1.30–8.54]		
IgG3 to MSP2 FC27		80.15/86.63	6.56	0.033	[0.52–12.60]		
IgGt to GLURP		72.44/85.39	12.05	0.040	[0.53–23.57]		
IgG1to GLURP		80.45/97.29	15.52	0.014	[3.17–27.87]		
IgG1 to MSP1	ACHP/ACLP	73.67/85.30	10.36	0.007	[2.93–17.78]		
IgG3 to MSP2 FC27		86.63/92.82	5.89	0.015	[1.14–10.64]		
IgG3 to MSP3		65.92/75.31	8.70	0.037	[0.52–16.87]		
IgGt to GLURP		85.39/96.07	8.70	0.037	[0.52–16.87]		
IgGt to MSP1	UMA/ACLP	73.97/84.56	9.18	0.045	[0.20–18.15]		
IgG1 to MSP1		74.48/85.30	9.84	0.014	[2.00–17.68]		
IgGt to MSP2 3D7		105.34/113.19	7.61	0.017	[1.35–13.85]		
IgG3 to MSP2 3D7		95.13/100.41	5.24	0.002	[1.89–8.59]		
IgGt to MSP2 FC27		79.88/97.56	14.89	0.002	[5.69–24.08]		
IgG3 to MSP2 FC27		80.15/92.82	11.07	<0.0001**	[5.76–16.39]	0.004	0.72
IgGt to GLURP		72.44/96.07	21.58	<0.0001**	[11.14–32.02]		
IgG1 to GLURP		80.45/103.04	19.25	0.001**	[8.09–30.39]	0.035	0.90
IgG3 to GLURP		59.47/82.20	20.43	0.002	[7.74–33.13]		

* The effect of malariometric status on IgG responses (IgGt: total IgG), determined by ANCOVA, was adjusted for age.

** Significantly different after adjustment for multiple tests (Bonferroni correction, threshold of significance: P = 0.002).

*** Logistic regression was applied for the analysis of associations with protection against malaria attacks, where status (UMA vs ACLP groups) was used as the dependent variable against the explanatory variables (IgG subtype responses (IgG3 to MSP2 FC27 and IgG1 to GLURP) and age) shown to be significantly associated by ANCOVA. OR values were assessed for 10 AU increased. Age was also associated with malaria protection in the analysis (p = 0.011, OR = 0.84).

Associations between antibody responses and protection against higher-grade parasitemia were assessed through comparison of IgG levels in the ACHP and ACLP. Here, the levels of IgGt directed to GLURP, of IgG1 to MSP1 and of IgG3 to both MSP2 (FC27 allelic form) and MSP3 were higher in the ACLP compared to the ACHP group (Table 3). Again, however, after multiple test adjustments only the difference in IgG1 to MSP1 remained with borderline statistical significance, and logistic regression was therefore not conducted.

The association between specific antibodies and the combined ability to protect against disease and high-grade parasitemia was assessed through comparison of the IgG levels in the ACLP and UMA groups. This analysis showed that the levels of IgGt directed to MSP1, to MSP2 (both allelic forms) and to GLURP, as well as of IgG1 to MSP1 and to GLURP and of IgG3 to both MSP2 (both allelic forms) and to GLURP were all higher in the ACLP compared with the UMA group. After multiple test adjustments, the higher levels of IgGt and of IgG1 to GLURP and of IgG3 to MSP2 (FC27 allelic form) remained statistically significantly different between the groups (Table 3). In the logistic regression model, the levels of IgG1 with specificity for GLURP (p = 0.035; OR = 0.90) and of IgG3 with specificity for MSP2 FC27 (p = 0.004; OR = 0.72) in the ACLP group were shown to be statistically significantly higher than those in the UMA group. Further analyses revealed no interaction between these two putatively protective IgG responses in the logistic regression model. Separately, and independently of the IgG associations described above, age was also found to be significantly higher in the ACLP group (p = 0.011; OR = 0.84).

Study participants' cumulated antibody-mediated recognition of multiple antigens at high levels was also found, independently of age, to be associated with the outcome of infection with *P. falciparum*. Here, logistic regression analyses revealed 3.6-fold greater odds (p = 0.0003) of being in the ACLP group versus the UMA group when responding strongly to 2 or more antigens, and 2.4-fold greater odds (p = 0.019) of an individual being in the ACLP group versus the ACHP group when responding strongly to 3 or more antigens.

### Parasite growth inhibitory properties of IgG

**Figure 4:**
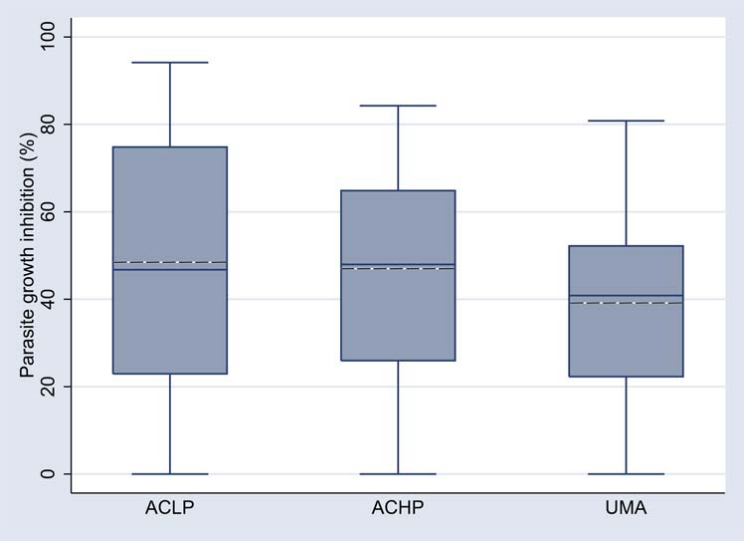
Parasite growth inhibitory capacity of purified IgG in groups segregated according to malariometric status. Data from IgG purified from a total of 75 plasma samples is illustrated for groups of children segregated according to their status as either low (ACLP, <2500 parasites/ul) or high (ACHP, ≥2500 parasites/ul) asymptomatic parasitemia carriers, or those with one or more malaria attacks (UMA, parasitemia plus fever) during 12 months' follow-up. 15 samples (5 matched trios) were excluded due to large variations between triplicate samples (coefficient of variation >30%). Box-whisker plots represent medians with 25^th^ and 75^th^ percentiles (boxes), and with 10^th^ and 90^th^ percentiles (whiskers), outliers as discrete dots. Doted lines represent the arithmetic mean of parasite growth inhibition.

**Figure 5:**
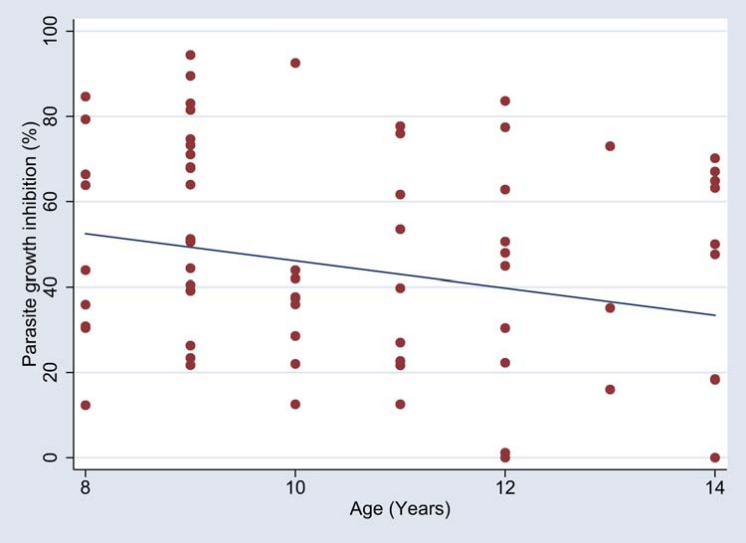
The association between parasite growth inhibitory activity of purified IgG and age. Parasite growth inhibitory (PGI) capacity of purified IgG plotted against age, with regression line fitted (using the regression fit command of STATA software). A significant difference in PGI (p = 0.03, Kruskall-Wallis test) was observed between groups segregated according to age (8–9 years vs 10–11 years vs 12–14 years).

The PGI of the matched (according to age, gender and area of residence) samples selected from the UMA, ACHP and ACLP groups did not differ significantly (Fig. 4), although the ACLP group had the highest mean level of PGI (48.4%), the ACHP group slightly lower (46.5%) and the UMA group the lowest (39.0%). In addition, the level of PGI decreased significantly as a function of increasing age (Fig. 5).

In further analyses, ELISA-detectable antibody levels in the original plasma were compared in the same set of samples segregated according to their PGI activity: a ‘high’ group (36 individuals) with PGI>50%, and a ‘low’ group (47 individuals) with PGI≤50%. The group with high PGI activity had, after correction for multiple comparisons, significantly higher levels of IgGt directed to MSP1 and to MSP2 (both allelic forms) than those with low PGI activity. Among the cytophilic subclasses, elevated IgG1 responses to MSP1 and IgG3 responses to MSP2 (both allelic forms) were associated with a higher capacity to inhibit parasite growth (Table 4). Anti-AMA1 IgG antibody responses were not associated with anti-malarial protection in the analyses of sero-epidemiological data, however significantly higher levels of total anti-AMA1 IgG, and particularly of anti-AMA1 IgG1 antibodies, were found in those with higher PGI capacity (Table 4).

**Table 4:** Comparison of IgG levels in groups of samples with high or low parasite growth inhibition (PGI).

	PGI≤50%*(n = 47)	PGI>50% (n = 36)	P**
IgG responses	Median of Ab responses in arbitrary units (5–95 percentile)		
MSP1			
IgGt	64.74 (3.72–110.13)	96.34 (29.41–112.93)	0.0005***
IgG1	65.65 (8.71–101.60)	97.73 (42.48–103.93)	0.0005***
MSP2 FC27			
IgGt	66.18 (12.90–114.07)	95.08 (38.04–136.91)	0.0015***
IgG3	72.11 (37.76–101.99)	90.46 (50.83–105.28)	0.0043
MSP2 3D7			
IgGt	108.56 (65.98–124.65)	118.92 (67.33–131.88)	0.0005***
IgG3	99.43 (64.40–105.12)	102.77 (79.83–108.13)	0.0086
MSP3			
IgGt	44.58 (0–100.31)	44.47 (0–99.94)	0.33
IgG3	66.51 (21.25–101.73)	64.64 (26.08–101.33)	0.77
GLURP			
IgGt	70.42 (4.91–135.36)	78.09 (23.27–135.14)	0.03
IgG1	76.79 (3.81–141.86)	97.97 (29.54–145.74)	0.04
IgG3	51.55 (0–131.74)	81.50 (0–132.37)	0.07
AMA1			
IgGt	89.86 (33.77–109.02)	104.50 (93.87–110.44)	0.0001***
IgG1	88.23 (13.67–103.28)	100.51 (89.25–104.91)	0.002***

* No significant difference in age was observed between the groups of samples showing high and low level of PGI (p = 0.33, Mann-Whitney).

** Non parametric Mann-Whitney test was used to compare IgG levels in the groups.

*** Significant p value after multiple test adjustment (Bonferroni correction).

## Discussion

In this study we used retrospective analyses to assess the associations between parasite-specific antibody activity and protection from malaria and/or high grade parasitaemia. Although therefore differing in design from more commonly-used prospective studies, precluding direct comparison of results, our findings do support the continued development of vaccines based on specific *P. falciparum* asexual stage antigens. A distinguishing feature of our study is that we combined quantification of IgG subclass responses to a panel of *P. falciparum* vaccine candidate antigens with determinations of functional antibody activity using purified IgG in a standardized *in vitro* assay of *P. falciparum* asexual stage growth inhibition. In addition, the continuous and close active surveillance of study participants we conducted over 1 year allowed for precise definitions of their clinical and parasitological phenotypes. The study therefore had sufficient power, using adjusted analyses where appropriate, to identify independent associations between anti-malarial protection and defined antibody responses. Of equal importance, we feel, is the fact that our findings emphasize the need to identify IgG subclass-specific responses in sero-epidemiological studies of this kind rather than simply total IgG.

Our data show that naturally-acquired clinical and parasitological immunity in children living in an area characterized by stable, highly seasonal transmission of *P. falciparum* malaria is associated specifically with (i) cytophilic IgG1 and IgG3 responses (the latter of borderline significance) to GLURP and (ii) IgG3 responses to MSP2, measured after the transmission season. We found, furthermore, an association of borderline significance between anti-parasite immunity and higher levels of IgG1 directed to MSP1. All of these associations, importantly, were independent both of each other and of age. The possible functional attributes *in vivo* of both the anti-MSP1 and -MSP2 responses were further emphasized by their associations with enhanced inhibition of parasite growth by purified IgG *in vitro*.

Despite their evident design differences, then, the results of our retrospective study are nevertheless consistent with those of other prospective sero-epidemiological studies that have shown associations between anti-malarial protection and IgG responses directed to either the GLURP R0 domain [27], [28], [29], [30], [31], [32] or to MSP2 [20], [28], [33], [34], [35], [36], [37], [38], although two very recent studies could not confirm all those findings [39], [40]. The reasons for discordant observations could be numerous, and most likely include specific differences in either technical aspects or study design, but we favor malaria transmission patterns, and thus primarily cumulative variations in the degree of exposure to particular antigens, as probably the strongest influence on outcomes [9]. One other recent study is notable in this context, since it reports findings that are in marked contrast to those of our own and others' [40]. That study, involving a Senegalese village population, reported that anti-MSP3 IgG3 responses alone - and none of the responses to any of the other antigens tested, including those assessed in the study presented here - were associated with anti-malarial protection. It should be stressed, however, that malaria in the area of that particular study village is both holoendemic and perennially transmitted, thus clearly distinguishing it from most other studies [40]. A recent Kenyan study also identified anti-MSP3 IgG responses as being strongly associated with protection, but this again concerned a population with distinctly different exposure to malaria compared to our own cohort [35]. We employed the same operating procedures (i.e. standardized protocols developed by the AfroImmunoAssay consortium) with a panel of antigens similar to that used by Nebié and colleagues in their study of Burkinabe children [31]. Malaria transmission in their study area is very similar to that in ours, and the studies were conducted in the same year (2003). Their findings may thus plausibly be compared directly with our own. They reported significant associations with anti-malarial protection for anti-GLURP (IgG3) and anti-AMA1 (IgG1) responses, but no such associations for either anti-MSP1 or -MSP3 IgG, while anti-MSP2 IgG responses were not assessed [31]. Although clearly not identical the broad similarities of the findings of the two studies are nevertheless striking, and indeed lend credence to GLURP's vaccine candidacy. The different outcome for anti-AMA1 IgG may reflect the inclusion of Burkinabe below 4 years of age, since seroconversion to AMA1 occurs substantially earlier in life than for other antigens, and responses to the latter may mask any effects of anti-AMA1 IgG in older individuals. On the other hand, another recent study that included children of a similar age-range to ours did identify associations between anti-malarial protection and anti-AMA1 IgG3, albeit in Papua New Guineans [41]. No plausible explanation is immediately obvious for the difference between the findings of the latter study and our own. Transmission-related differences may be important, possibly including the unknown effects of exposure to both *P. falciparum* and *P. vivax*.

The accurate phenotypic segregation of our study participants allowed us to identify IgG responses with putatively varying functional attributes. Anti-parasite activity appeared to be particularly associated with IgG1 directed to MSP1, since the magnitude of this response distinguished those (the ACLP group) able to control parasitemia above a defined threshold from those (the ACHP group) that could not. This finding is consistent with the results of other sero-epidemiological studies of this molecule, some of which also identified an association of anti-MSP1 IgG with control of high-grade parasitaemia [39], [42], [43], [44], [45], [46]. As mentioned above, our observation of stronger parasite growth inhibitory activity *in vitro* in samples containing the highest levels of anti-MSP1 IgG1 lend strong support to the notion that such antibodies indeed have a functional role *in vivo*. Although displaying only non-significant trends, differences in the levels of anti-MSP2 IgG3 suggested some degree of association with clinical immunity (UMA vs ACHP groups, Table 3), but, more plausibly, when assessing the sero-epidemiological together with the functional *in vitro* findings, these antibodies appear to confer substantial protection against high parasitaemia. A logical conclusion would therefore be that cytophilic anti-MSP1 and anti-MSP2 IgG function in an additive or even synergistic way to prevent parasite multiplication.

We observed markedly contrasting associations for IgG responses directed to GLURP compared with those directed to AMA1. On the one hand, the sero-epidemiological analyses revealed that cytophilic anti-GLURP IgG activity was strongly associated with anti-malarial protection (Tables 2 & 3), but this was not the case for anti-AMA1 IgG, while higher levels of the latter antibodies were associated with functional activity *in vitro* but no such association was detectable for anti-GLURP IgG (Table 4). It is known that IgG responses to AMA1 are acquired rapidly in early life, as mentioned above, and most sero-epidemiological studies that have revealed anti-malarial protective associations for anti-AMA1 IgG have included children below the age range of those in our study, possibly, therefore, offering an explanation for the different outcomes [28], [31], [35], [38], [41]. For GLURP, pre-existing knowledge suggests an explanation for the apparent lack of anti-GLURP IgG activity in the functional assay. This explanation rests on the proposed mechanism of cooperation between monocytes and cytophilic IgG in mediating parasite growth inhibition [2], [7], and the evidence for a functional role of anti-GLURP antibodies in such a process [47], [48]. Our own preliminary data show appreciable (up to 52%) monocyte-mediated parasite growth inhibition by affinity-purified anti-GLURP R0 antibodies derived from a pool of six plasma samples with high IgG responses against GLURP R0 randomly selected from children in the study described here (D. Courtin & M. Theisen, unpublished observations). Anti-GLURP antibodies thus seem not to exhibit the type of direct anti-parasite effects that the growth inhibition assay used here aims to quantify, but rather act indirectly through opsonic interactions with merozoites and monocytes.

Finally, some aspects of the growth inhibition assay employed here are worthy of mention. Despite using purified IgG - effectively precluding any influence of anti-malarial metabolites or other potentially parasitistatic molecules in the *in vitro* assay - we did not find significant differences in functional activity between the groups segregated on the basis of clinical and parasitological phenotypes. This is in line with the results of studies that used either dialysed or whole serum rather than purified IgG, but that nevertheless found no association between the amounts of growth-inhibitory antibodies detectable *in vitro* and reduced risk of malaria [49], [50], [51], but contrasts with those of another recent study from Kenya [52]. Whether the outcome in this context was affected by our use of a standard concentration (10 mg/ml) of IgG - well below the normal level to be found in African children's blood - in the assay procedure we adopted is not known. One consistent finding in this and the other recently published studies that have used a broadly comparable *in vitro* assay concerns the age-related decline in growth inhibitory activity [50], [52]. The explanation for this inverse association remains obscure, but one implication is that the naturally-acquired protection enjoyed by older age-groups relies on alternative parasite growth inhibitory mechanisms. In any case these findings serve to reiterate the need for development and standardization of adequate and appropriate *in vitro* assays that faithfully reflect *in vivo* antibody function.

In summary, the results of this study clearly highlight the role of IgG antibody subclass responses in acquired anti-malarial immunity. On the basis of the data presented, we conclude that naturally-acquired immunity to malaria, in the setting described, relies on the presence of a constellation of cytophilic IgG antibodies, displaying differing specificities and probably having disparate functional attributes, but that may operate in concert *in vivo* to suppress parasitaemia to levels below those causing disease. We interpret the results as providing strong support for the development of a vaccine designed to elicit antibody responses simultaneously to multiple asexual stage antigens of *P. falciparum*.
